# The Development of the Pacific Ocean Shelf Tracking Project within the Decade Long Census of Marine Life

**DOI:** 10.1371/journal.pone.0018999

**Published:** 2011-04-28

**Authors:** George D. Jackson

**Affiliations:** Department of Health Sciences, Weimar College, Weimar, California, United States of America; National Institute of Water & Atmospheric Research, New Zealand

The Pacific Ocean Shelf Tracking (POST) project was established as one of the 17 projects of the decade-long Census of Marine Life. Its initial purpose was to improve our understanding of the distribution and life history of salmon on the continental shelf. POST was made possible by developments in acoustic technology which resulted in miniaturization of acoustic tags along with the creation of passive acoustic receivers which can be deployed in ‘listening curtains’ along the seafloor. The development of receivers with an acoustic modem has greatly facilitated the practicality of deploying marine lines, as data can now be uploaded to ship remotely. The POST array, which now spans over 3,000 km from California, through British Columbia, to Alaska, is composed of over 400 receivers in 10 ocean lines and strategic regions within rivers. The POST array and the associated database serve as a research tool for answering critical questions on ecology and marine resource management. Valuable data has been collected on salmonids and other marine species, and the combination of POST technology with other molecular and physiological tools is already revealing important clues in mortality and migration behaviours. POST has created a proof-of-concept, continental-scale marine tracking array and has served as a valuable pilot project despite the fact that it didn't realize its full potential envisioned at the beginning of the Census of Marine Life. POST has however served as a flagship model for developing large scale arrays in other regions of the world through the international Ocean Tracking Network.

## Background to POST and the Census of Marine Life

The Pacific Ocean Shelf Tracking (POST) project is one of 17 projects of the decade-long Census of Marine Life (CoML). The overall goal of the CoML was to: “assess and *explain* the changing *diversity, distribution and abundance* of marine *species*, from the past to the present, and project future marine life” [1] (authors' italics). The POST project was established to focus primarily on the present distribution of marine continental shelf species. Research undertaken using the POST array however is ongoing and dynamic with researchers obtaining valuable information on how the distribution of marine (or diadromous) organisms changes over the course of their life cycle [2]. POST helps answer questions on why and how aquatic organisms move in relation to their changing environment. POST is a resource available to any researcher to explore the dynamics of the movement and distribution of a continental shelf species within the Northeast Pacific.

The papers in this special collection represent work of independent investigators using the POST array to advance marine science. These papers offer examples of a number of studies on a variety of species in different environments. In all cases the results have revealed things that would have been difficult to obtain by other means, yet results and conclusions remain those of the investigators and not the POST Project.

## POST Technology

Traditional tagging involved attaching a non-electronic tag to a fish or other marine organism and could only provide a relatively crude and inaccurate picture of animal movement. In essence, older techniques only provided information on the release and capture points, with no information on movement between these two points. New electronic and computerized tags have since been developed, which are now providing us with a plethora of information on marine animal movements. Our ability to track the movements of marine organisms continues to move forward rapidly with the development of many different technologies. Satellite tags are now being deployed which can track megafauna for vast distances across the oceans [3], [4], and archival tags also collect amazing detail on animal movement particularly in relation to oceanography [5], [6], but that detail can only be obtained if the tag can be retrieved. For larger marine organisms which do not come to the surface, pop-up satellite archival tags (PSAT) have been developed which incorporate archival position data relayed to a base station via satellite after the tag pops off the organism at a set time [5], [7], [8].

Smaller passive integrated transponder (PIT) tags [9], [10] have the advantage of being very tiny and not requiring batteries, however, the disadvantage is that in order to detect PIT tagged organisms they need to pass very close to a reader or antenna. This works well in areas where for example fish need to negotiate around a dam through a narrow passage, or a bottleneck in a stream, but PIT tag technology cannot be effectively used in open ocean portions of the continental shelf environment.

During the development of various tracking technologies, there clearly was a need to track continental shelf organisms too small to carry satellite tags, which may not be recaptured to obtain movement data recorded in archival tags, and that are not able to be detected within the necessary proximity of PIT receivers. Acoustic tag technology has filled this important niche for tracking marine, anadromous and even freshwater organisms.

**Figure 1:**
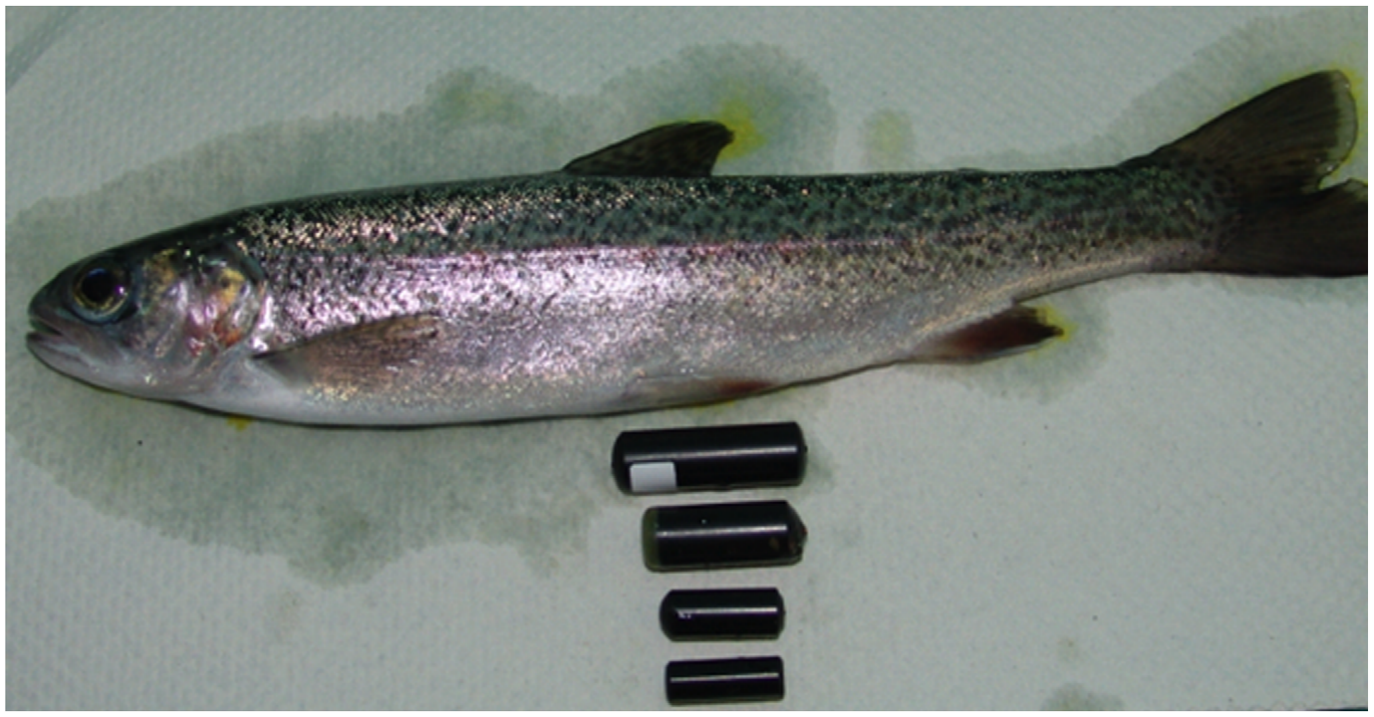
A salmon smolt shown with the variety of acoustic tags and how the size of acoustic tag has become reduced as the technology advanced.

Acoustic tracking was facilitated by the development of uniquely-coded tags which provide a means to track individuals over time. As the technology progressed these tags became small enough to be implanted in animals the size of salmon smolts (Figure 1). Each miniature tag periodically broadcasts its unique acoustic signal. In order for the organism to be tracked, the code needs to be detected by a passive receiver deployed on the ocean floor or towed by a vessel. Acoustic tags have been developed in a variety of sizes (some examples shown in Figure 1) and power output, enabling considerable flexibility in use. There are obvious tradeoffs between battery power, tag power and life span. Studies on larger organisms can take advantage of very large tags with extended battery life, while smaller organisms require much smaller, less powerful tags with a shorter life span. However, tag broadcast timing can be adjusted to extend battery life and tags can even be programmed to sleep and turn on at a later date. Thus there is considerable scope to customize tag parameters to fit the constraints and needs of a particular study.

**Figure 2:**
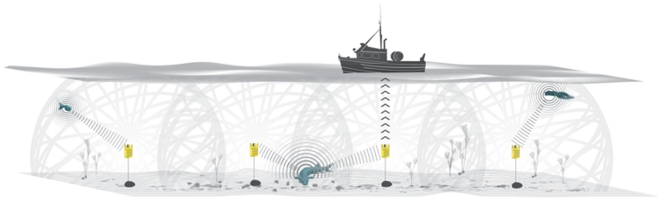
A schematic of how post works. Four POST receivers are shown anchored to the sea bottom and enclosed within the flotation collars. Two tagged fish and a tagged squid are depicted being detected by the receivers while the ship is depicted uploading POST data from a receiver via an acoustic modem. The grey lines represent the spheres of detection range of each receiver showing overlap in detection range between adjacent receivers (hence the two receivers are simultaneously downloading data from a tagged fish that is situated in the region of detection range overlap).

The passive receiver is equipped with an omnidirectional hydrophone that listens and records the passing of any tagged marine organism in its vicinity [11], [12]. While the detection radius of the receiver varies depending on conditions within the water column and ambient noise, in the open water the range can be on the order of 100 m to 1000 m, depending on the power of the transmitting tags (Figure 2).

**Figure 3:**
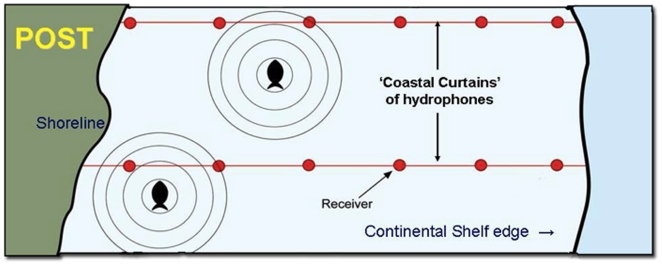
Diagrammatic representation of continental shelf POST listening lines. The lines of receivers run from the shoreline to the edge of the continental shelf. Tagged fish swimming alongshore send out a unique acoustic code which is then detected by receivers which record the time and date a fish passes the receiver.

In order to quantitatively track marine organisms across a large distance using acoustic technology, there was a need to be able to create lines of receivers which would record the time and date that an organism crossed the line (Figure 3). This was first successfully carried out with Atlantic salmon smolts in eastern Canada, where lines of receivers were used to record smolt movements during their migration [13]. This early study provided the ‘proof-of-concept’ for producing a larger scale array on the west coast of North America, which grew into the POST project.

**Figure 4:**
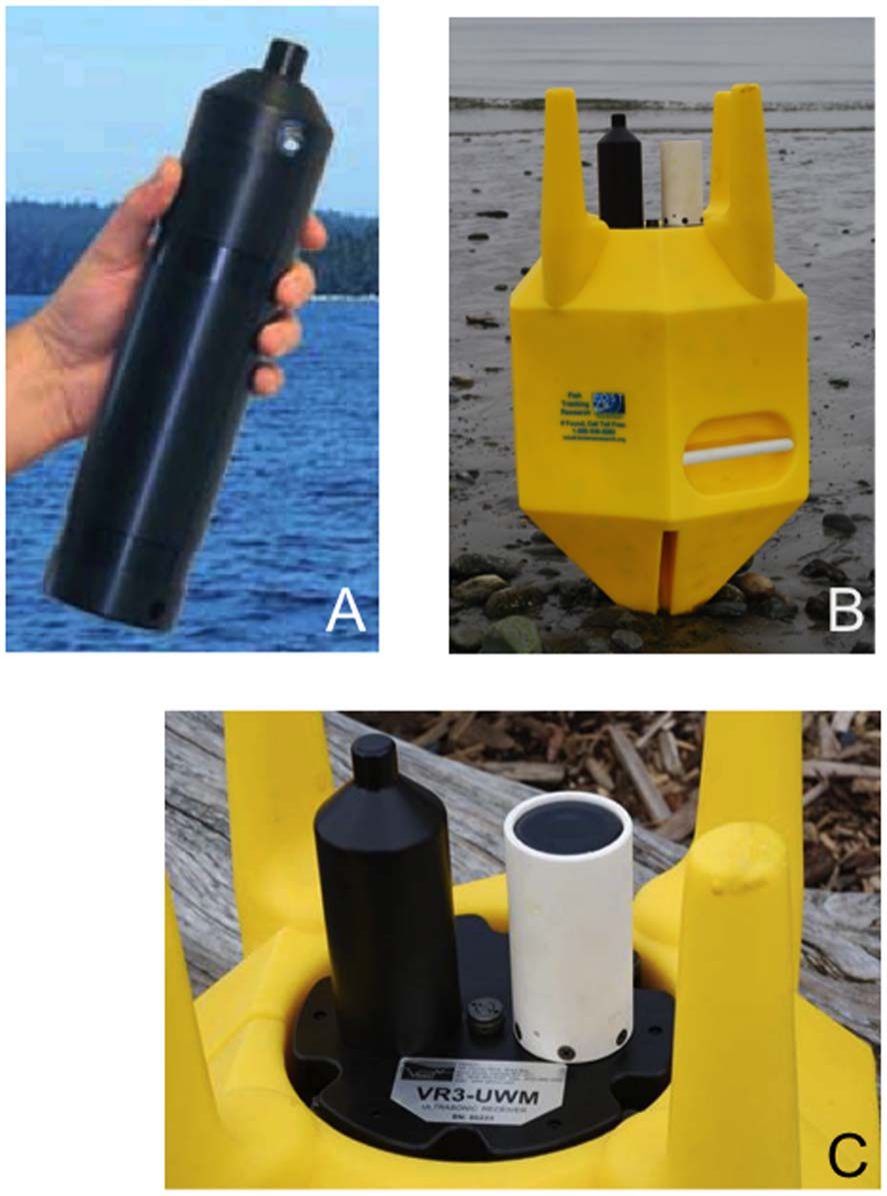
The development of acoustic receivers. (A) the VR2 acoustic receiver that is inexpensive but requires the retrieval from the seafloor to download the data (B) the VR3 receiver enclosed in the flotation collar that keeps the receiver vertical in the water column and protects the receiver from trawler damage (C) A close-up of the top of the VR3 receiver enclosed in the flotation collar with the black receiver on the left and the white acoustic modem (for remote data upload) on the right.

The receivers used in the above-mentioned Atlantic Salmon study [13], and the initial lines of the POST array, were essentially data logging devices which kept records of passing tagged organisms in an onboard memory. Theses receivers (Vemco VR2, Figure 4A) have a battery life of approximately 15 months and have to be retrieved from the seafloor in order to download the data. Thus information on fish passing an acoustic line could not be obtained until after a period of time, and with considerable effort and often expense in retrieving the VR2 receivers from the seafloor. The continental shelf scale of the POST project required a more efficient and cost effective way of gathering data on a more regular basis. This led to the development of an acoustic receiver integrated with an acoustic modem (Vemco VR3, Figure 4B,C). This next generation unit had a larger battery pack that could potentially stay on the ocean bottom for 5–7 years and important data could be uploaded periodically from the seafloor directly to a vessel on the surface. These newer units have worked very efficiently within the POST array and have helped to automate the process of data upload, as well as greatly reducing costs by negating the need to annually retrieve lines of deepwater units from the ocean floor to obtain data.

## The Continental Array

**Figure 5:**
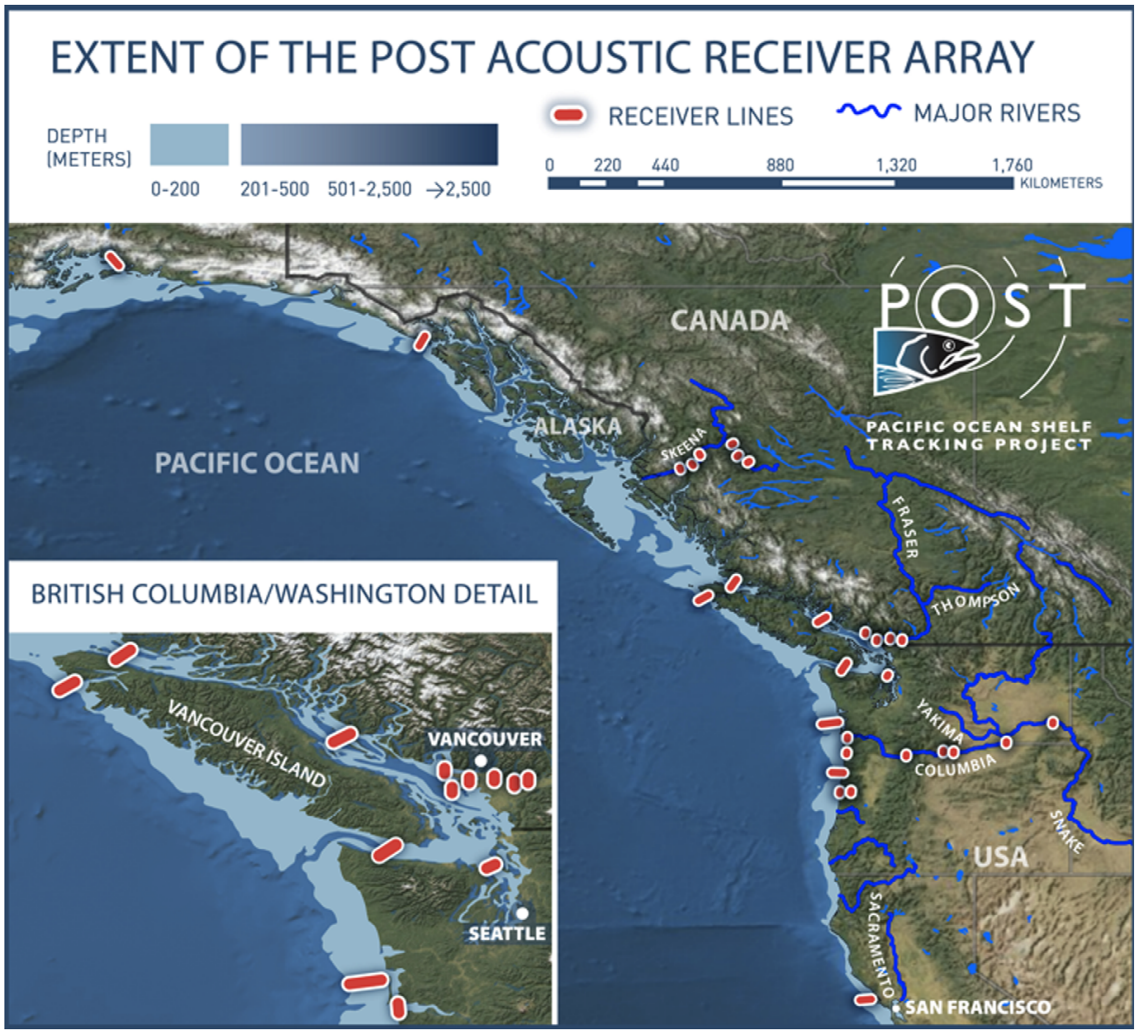
The extent of the POST array in 2009 showing receiver lines extending from California to Alaska and along several major rivers.

The POST array spans over 3,000 km along the Northeast Pacific coast and includes more than 400 acoustic receivers. There are 10 marine lines situated from Port Gravina, Prince William Sound, Alaska in the north, to Point Reyes, California in the south (Figure 5).

The broad expanse of the array can help address questions on large-scale movement and migration. However, integrating the broad system with local arrays can provide a much richer data set on movement and mortality. The high performance of the system in both fresh and marine water allows use of the POST array, together with local or regional arrays, in many of the large river systems and their estuaries on the west coast of North America, including the Fraser and Skeena Rivers in Canada, and the Columbia and Sacramento Rivers in the USA.

The original Census of Marine Life vision for POST was a continental-scale array stretching from the Baja Peninsula to the Bering Sea. While POST has not yet achieved this envisioned scale, it continues to expand and its success and usefulness have demonstrated a feasibility that is applicable along continental shelves around the world.

## POST Data

POST is a powerful observing tool that generates valuable data about coastal organisms, contributing to greater understanding and improved management and conservation of important marine resources. The value of POST has been demonstrated by a variety of studies which already have revealed important and sometimes surprising results. However, the power of POST is still largely untapped for many species. The potential for the continental array to be a significant tool for ongoing science is immense and only limited by the needs and imagination of the community of scientific users. The future success of POST is enhanced by the ability to follow marine organism movement and migration patterns in an otherwise opaque environment, and to track these organisms between freshwater and marine environments. Thus, POST will continue to be critical to salmonid and other marine ecologists who have ongoing need to differentiate movement and migration behaviour between both abundant and endangered populations. The high detection efficiency of the POST lines will enable future studies on movement and even survival to be carried out with relatively small sample sizes, which will greatly reduce cost and pressure on endangered stocks [14], [15]. One study using the POST array [15] provided strong corroboration that survival of some juvenile salmon was actually higher in the freshwater sector of the migration than in the ocean, even though the migrating smolts had to negotiate around a number of dams. This result challenged some of the conventional wisdom of salmon ecology [16]. It is expected that POST will continue to play a cutting-edge role to help answer these ecological and sometimes controversial questions regarding marine ecology and survival.

New applications for POST technology continue to be developed (see POST publication list http://www.postcoml.org/page.php?section=community&page=publications). However, the full potential of the POST array has yet to be realized for many species. To date 18 species have been tracked including: green sturgeon, white sturgeon, six-gill shark, seven-gill shark, salmon shark, spiny dogfish, lingcod, jumbo squid, market squid, spotted ratfish, cutthroat and steelhead trout, dolly varden, black rockfish and chum, coho, sockeye and chinook salmon. This work has been carried out by over 45 researchers using POST-generated data.

Studies focusing on simple movement patterns have already revealed marked international movement for green sturgeon, [17] which are pointing to improved international conservation and the value of cross-border cooperative management of fish stocks. These results revealed that even relatively simple movement studies can have profound ramifications. However, POST also lends itself to more sophisticated studies where data from acoustic tracking can be used in concert with other new techniques such as genomic and physiological tools [18].

Developing these new applications will further demonstrate how POST can continue to serve as an experimental platform for testing theories through integrated studies. It is expected that POST will serve as an important monitoring and experimental tool for a suite of species whose movement and migration behaviour may be affected by changing ocean conditions.

POST is not only an observational array, but also incorporates an international database. Thus in the future, while it may be impossible to detect trends in behavior from climate change or changing ocean conditions in any single study, large scale meta-analyses could reveal broader patterns in changing behaviour. The POST database will therefore help to advance science through large-scale and international data sharing and by linking the global community through the Census of Marine Life's Ocean Biogeographic Information System (OBIS) (www.iobis.org).

## Limitations of POST

Now that Census of Marine Life has concluded it is useful to examine what POST did and did not achieve. How close did POST get to achieving its original goal of an extensive array consisting of many lines of receivers from Baja to Bering [19]? While the current geographical spread of receivers ranges from California to Alaska, concentration of the receiver lines is minor compared to the original vision. The greatest concentration of lines was in the Salish Sea region. To achieve its full potential requires considerable funding on an international scale. This was not achieved during the decade-long Census, and the cost to get to the end result of the original vision would be substantial. The price of acoustic receivers along with the ship time for deployment and data upload also required considerable investment. The lack of a greater concentration of lines has limited our ability to better interpret movement and migration patterns in high resolution, and many questions remain on where exactly mortality takes place.

The cost for the tags has also limited the number of individual fish that could be tagged, as researchers’ budgets are limited. While tag size has decreased over the decade of the Census, physical tag size continues to limit the size of fish that can be studied.

However, despite the limitations, POST provided a proof-of-concept and the development of necessary protocols, which has greatly advanced acoustic research. Fish can now have tags surgically implanted with extremely low mortality. Information on movement, migration and mortality has been collected that would be difficult or impossible to collect by any other means.

## The Future and the Globalization of POST

**Figure 6:**
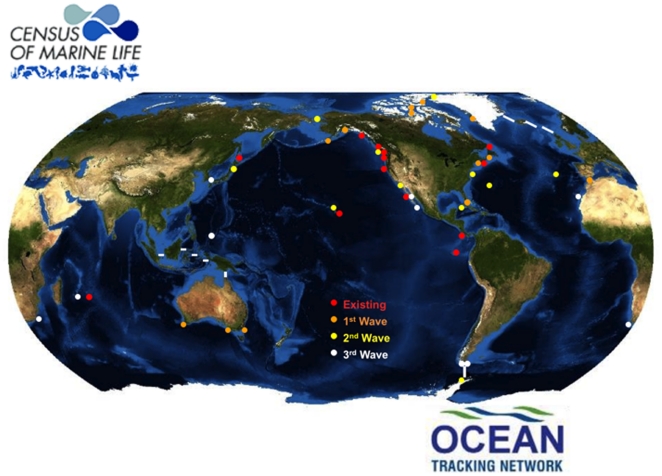
The proposed extent of POST-like arrays currently and proposed to be deployed by the Ocean Tracking Network around the world. The proposed deployments will occur in three phases or waves.

As technology advances, the equipment for acoustic telemetry continues to become more miniaturized, cheaper and more powerful. The future of POST not only lies in enhanced studies and collaborations in the Northeast Pacific, but can serve as a model for similar projects around the world. The Ocean Tracking Network has used POST as a flagship program to extend the POST concept globally (Figure 6) and to ultimately incorporate global arrays into the UN Intergovernmental Oceanographic Commission's Global Ocean Observing System (GOOS) [12]. The future vision of POST is an expanded array from Bering to Baja with considerably more infill of listening lines, so it is possible to better compartmentalize the continental shelf and pinpoint both congregation hotspots and areas of mortality. Globalization through OTN will also help to drive the technology forward so acoustic tags might ultimately be combined with archival tags, and new generation ‘business card’ tags that will communicate with each other. These new tags could in turn download their data sets to new generation receivers in the future POST array.

## Conclusion

The papers presented in this special POST collection give examples of how new technology is providing valuable information for resource and ecology management. These publications also provide inspiration for future studies on topics such as the relationship of animal behavior to changing ocean conditions. The information that POST can provide was not obtainable even a decade ago. The development of new tags and the expansion of tagging studies will continue to drive the research forward. Given the surprises that have already surfaced from research using POST, it is expected that ongoing and future studies will reveal even more startling and highly relevant data.
